# P-1446. Durable RSV-Specific CD4⁺ T-Cell Responses Following mRNA-1345 Vaccination in Adults at Increased Risk of Lower Respiratory Tract Disease Due to RSV

**DOI:** 10.1093/ofid/ofaf695.1632

**Published:** 2026-01-11

**Authors:** Erick F Mayer, Asefa Mekonnen, Ann R Falsey, Cameron R Wolfe, Christina Grassi, Avi Collins, Anisha Mannan, Md Hasan, Hsiaohsuan Kuo, Shannon McGrath, Archana Kapoor, Jenni Mou, Xiaolin Chang, Xing Chen, Lan Lan, Honghong Zhou, Sonia K Stoszek, Eleanor Wilson, Jaya Gowami, Rituparna Das, Frances Priddy

**Affiliations:** Moderna, Inc., Cambridge, MA; Velocity Clinical Research, Rockville, Maryland; University of Rochester School of Medicine, Rochester, New York; Duke University, Durham, NC; Moderna, Inc., Cambridge, MA; Moderna, Inc., Cambridge, MA; Moderna, Inc., Cambridge, MA; Moderna, Inc., Cambridge, MA; Moderna, Inc., Cambridge, MA; Moderna, Inc., Cambridge, MA; Moderna, Inc., Cambridge, MA; Moderna, Inc., Cambridge, MA; Moderna, Inc., Cambridge, MA; Moderna, Inc., Cambridge, MA; Moderna, Inc., Cambridge, MA; Moderna, Inc., Cambridge, MA; Moderna, Inc., Cambridge, MA; Moderna, Inc., Cambridge, MA; Moderna, Inc., Cambridge, MA; Moderna, Inc., Cambridge, MA; Moderna, Inc., Cambridge, MA

## Abstract

**Background:**

Cell-mediated immune responses likely play a key role in durable protection against respiratory syncytial virus (RSV). Adults with certain medical conditions (eg, chronic lung disease, cardiovascular disease, diabetes) are at increased risk of RSV-associated lower respiratory tract disease (RSV-LRTD). We report T-cell responses following a single dose of mRNA-1345 in immunocompetent 18-59-year-old adults with such risk factors.

**Figure 1.**
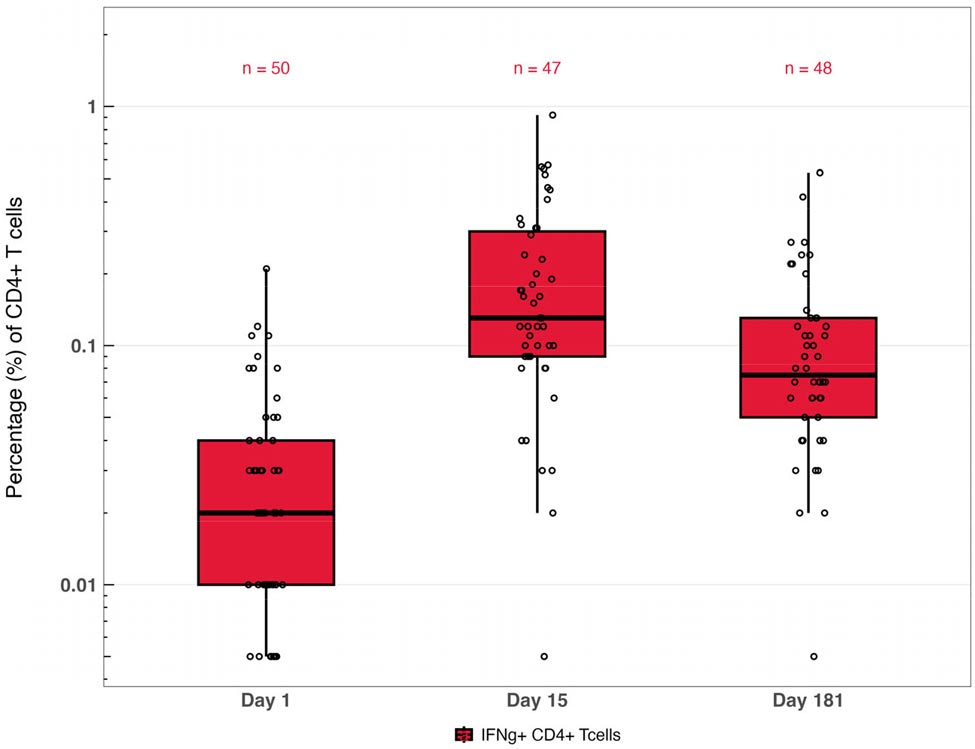
RSV PreF-specific CD4+ Th1 Responses (IFNγ+) Through 6 Months After Vaccination With mRNA-1345 (50 µg) in Adults Aged 18-59 Years

**Methods:**

Adults 18-59 years with ≥1 high-risk condition for RSV-LRTD received a single 50-μg dose of mRNA-1345 in an ongoing phase 3 trial (NCT06067230). Peripheral blood mononuclear cells were optionally collected from a subset of participants at baseline, Day 15, and Day 181. RSV-F (prefusion conformation) T-cell responses were assessed; CD4+ and CD8+ cells were evaluated with intracellular cytokine staining for Th1 (IFN, TNF, IL2) or Th2 (IL4, IL5, IL13) responses, in addition to other functional markers (ie, CD40L, a marker of B cell co-stimulation). Subgroup analyses were conducted for participants aged 18-49 and 50-59 years.

**Results:**

Fifty participants had PBMCs collected (18-49 years, n=21; 50-59 years, n=29). At baseline, RSV preF-specific CD4+ Th1 responses (IFNγ+) were low. After mRNA-1345, IFNγ+ CD4+ Th1 responses increased substantially by Day 15 and remained elevated through Day 181 (Figure). Polyfunctional CD4+ Th1 cells (expressing ≥2 or ≥3 cytokines) followed a similar trajectory. IFNγ+ CD8+ T-cell responses were also present at Day 15. CD4+ Th2 responses were minimal and transient on Day 15 and returning to baseline levels by Day 181; no polyfunctionality was observed. Responses were consistent between age subgroups.

**Conclusion:**

Vaccination with mRNA-1345 elicited Th1-biased CD4+ T-cell responses in adults aged 18-59 years with medical risk factors for severe RSV-LRTD. CD8+ T-cell responses were also observed. CD4+ responses were robust, persisted for 6 months post-vaccination, and were consistent across age subgroups. This demonstrates the ability of mRNA-1345 to induce cellular immunity in younger, vulnerable populations, with durable responses which may be important for prevention of severe disease over time.

**Disclosures:**

Erick F. Mayer, MD, Moderna, Inc.: Employee of Moderna, Inc.|Moderna, Inc.: Stocks/Bonds (Public Company) Ann R. Falsey, MD, ADMA Biologics: Advisor/Consultant|ADMA Biologics: Honoraria|AstraZeneca: Advisor/Consultant|AstraZeneca: Grant/Research Support|AstraZeneca: Honoraria|CynaVac: Grant/Research Support|GSK: Advisor/Consultant|GSK: Honoraria|Merck: Advisor/Consultant|Merck: Honoraria|Moderna, Inc.: Advisor/Consultant|Moderna, Inc.: Grant/Research Support|Moderna, Inc.: Honoraria|Pfizer: Grant/Research Support|Sanofi Pasteur: Advisor/Consultant|Sanofi Pasteur: Honoraria Christina Grassi, MD, Moderna, Inc.: Employee of Moderna, Inc.|Moderna, Inc.: Stocks/Bonds (Public Company) Avi Collins, BScN, Moderna, Inc.: Former Moderna, Inc. Employee|Moderna, Inc.: Stocks/Bonds (Public Company) Anisha Mannan, MS, Moderna, Inc.: Employee of Moderna, Inc.|Moderna, Inc.: Stocks/Bonds (Public Company) Md Hasan, PhD, Moderna, Inc.: Employee|Moderna, Inc.: Stocks/Bonds (Public Company) Hsiaohsuan Kuo, PhD, Moderna, Inc.: Employee|Moderna, Inc.: Stocks/Bonds (Public Company) Shannon McGrath, MS, Moderna, Inc.: Employee|Moderna, Inc.: Stocks/Bonds (Public Company) Archana Kapoor, PhD, Moderna, Inc.: Employee of Moderna, Inc.|Moderna, Inc.: Stocks/Bonds (Public Company) Jenni Mou, PhD, Moderna, Inc.: Employee of Moderna, Inc.|Moderna, Inc.: Stocks/Bonds (Public Company) Xiaolin Chang, PhD, Moderna, Inc.: Moderna, Inc. employee|Moderna, Inc.: Stocks/Bonds (Public Company) Xing Chen, Sc.D., Moderna, Inc.: Moderna, Inc. employee|Moderna, Inc.: Stocks/Bonds (Public Company) Lan Lan, PhD, Moderna, Inc.: Employee of Moderna, Inc.|Moderna, Inc.: Stocks/Bonds (Public Company) Honghong Zhou, Ph.D., Modena, Inc.: Employee of Moderna, Inc.|Modena, Inc.: Stocks/Bonds (Public Company) Sonia K. Stoszek, PhD, Moderna, Inc.: Employee of Moderna, Inc.|Moderna, Inc.: Stocks/Bonds (Public Company) Eleanor Wilson, MD, MHS, Moderna, Inc.: Employee of Moderna, Inc.|Moderna, Inc.: Stocks/Bonds (Public Company) Jaya Gowami, MD, Moderna, Inc.: Employee of Moderna, Inc.|Moderna, Inc.: Stocks/Bonds (Public Company) Rituparna Das, M.D., Moderna, Inc.: Employee|Moderna, Inc.: Stocks/Bonds (Public Company) Frances Priddy, MD, MPH, Moderna, Inc.: Employee of Moderna, Inc.|Moderna, Inc.: Stocks/Bonds (Private Company)

